# Early identification and severity prediction of acute respiratory infection (ESAR): a study protocol for a randomized controlled trial

**DOI:** 10.1186/s12879-022-07552-7

**Published:** 2022-07-20

**Authors:** Guanmin Yuan, Hongyu Wang, Yuanhan Zhao, Enqiang Mao, Mengjiao Li, Ruilan Wang, Fangqing Zhou, Shanshan Jin, Ziqiang Zhang, Ke Xu, Jinfu Xu, Shuo Liang, Xiang Li, Lijing Jiang, Lu Zhang, Jieyu Song, Tao Yang, Jinxin Guo, Haocheng Zhang, Yang Zhou, Sen Wang, Chao Qiu, Ning Jiang, Jingwen Ai, Jing Wu, Wenhong Zhang

**Affiliations:** 1 Department of Infectious Diseases, Shanghai Key Laboratory of Infectious Diseases and Biosafety Emergency Response, National Medical Center for Infectious Diseases, Huashan Hospital, Fudan University, No. 12 Middle Urumqi Road, Jing’an, Shanghai, 200040 China; 2 Departments of Emergency, Ruijin Hospital, School of Medicine, Shanghai Jiaotong University, Shanghai, China; 3 Department of Critical Care Medicine, Shanghai General Hospital, School of Medicine, Shanghai Jiaotong University, Shanghai, China; 4 Department of Infectious Disease, Tongji Hospital, School of Medicine of Tongji University, Shanghai, China; 5 Department of Respiratory and Critical Care Medicine, Tongji Hospital, School of Medicine of Tongji University, Shanghai, China; 6 Department of Respiratory and Critical Care Medicine, Shanghai Pulmonary Hospital, Tongji University School of Medicine, Shanghai, China; 7 Department of Critical Care Medicine, Minhang Hospital, Fudan University, Shanghai, 201199 China; 8 National Clinical Research Center for Aging and Medicine, Huashan Hospital, Fudan University, Shanghai, China; 9 Key Laboratory of Medical Molecular Virology (MOE/MOH), Shanghai Medical College, Fudan University, Shanghai, China

**Keywords:** Acute respiratory infection, mNGS, Multiplex PCR, Randomized controlled trials

## Abstract

**Background:**

The outbreak of SARS-CoV-2 at the end of 2019 sounded the alarm for early inspection on acute respiratory infection (ARI). However, diagnosis pathway of ARI has still not reached a consensus and its impact on prognosis needs to be further explored.

**Methods:**

ESAR is a multicenter, open-label, randomized controlled, non-inferiority clinical trial on evaluating the diagnosis performance and its impact on prognosis of ARI between mNGS and multiplex PCR. Enrolled patients will be divided into two groups with a ratio of 1:1. Group I will be directly tested by mNGS. Group II will firstly receive multiplex PCR, then mNGS in patients with severe infection if multiplex PCR is negative or inconsistent with clinical manifestations. All patients will be followed up every 7 days for 28 days. The primary endpoint is time to initiate targeted treatment. Secondary endpoints include incidence of significant events (oxygen inhalation, mechanical ventilation, etc.), clinical remission rate, and hospitalization length. A total of 440 participants will be enrolled in both groups.

**Discussion:**

ESAR compares the efficacy of different diagnostic strategies and their impact on treatment outcomes in ARI, which is of great significance to make precise diagnosis, balance clinical resources and demands, and ultimately optimize clinical diagnosis pathways and treatment strategies.

*Trial registration* Clinicaltrial.gov, NCT04955756, Registered on July 9th 2021.

## Background

Acute respiratory infection (ARI) is one of the major diseases that threatens human health worldwide, with high morbidity, high severity and high medical costs [1]. COVID-19 [2] in 2019 and the H1N1 influenza in 2009 [3] have imposed enormous healthcare burden and economic loss worldwide. According to World Health Organization (WHO), lower respiratory tract infections (LRTI) are the fourth leading cause of death globally in 2019 [4]. Respiratory tract infections can be caused by a wide spectrum of pathogens including bacteria, viruses, fungi, mycoplasma, chlamydia, etc., with similar clinical manifestations but completely different treatment. Besides, colonization and complex infection make it more difficult to make accurate diagnosis. Once diagnosed or treated improperly, severe pneumonia will develop, which is associated with high mortality and various complications [5].

Early and precise diagnosis for respiratory pathogens is of great significance for making appropriate treatment strategies, saving healthcare resources and reducing unnecessary use of antibiotics. The gold standard is culture or isolation from respiratory specimen [6], which is time-consuming along with low positivity. Nowadays, emerging technologies, such as multiplex polymerase chain reaction (PCR) and metagenomic next-generation sequencing (mNGS), have become more and more widely used in the rapid detection of respiratory tract infections. Multiplex PCR is relatively cost-saving and can report results within hours [7], which has become the most attractive test method and currently the most commonly used method in clinical laboratory [8]. Filmarray respiratory panel (FA-RP) is featured as automated sample preparation, nucleic acid extraction and nested multiplex PCR detection, which can detect more than 20 pathogenic microorganisms in one test, including 17 respiratory viruses and 3 atypical pathogens [7, 9]. QIAstat-Dx RP is another full-automated multiplex real-time PCR test for identifying common respiratory viral and bacterial pathogens within 1 h [10]. Although diagnostic performance of multiplex PCR has been proved to be satisfying [11], detection spectrum of microorganisms is still limited, especially in rare or emerging pathogens [12]. mNGS can identify pathogens without subjective bias within 48 h [13, 14], but is more time-consuming, more expensive and requires well-equipped laboratory to perform.

Although a variety of diagnosis tools have been applied clinically, there still lacks consensus on clinical diagnosis pathway for ARI. The impact of multiplex PCR and mNGS on prognosis of ARI is still unclear. Therefore, based on mNGS and multiplex PCR detection, the study proposes a multicenter, open, randomized controlled clinical trial to compare the efficacy of different diagnostic strategies and its impact on prognosis for ARI, which ultimately aims to optimize the diagnostic pathways and treatment strategies.

## Methods

### Study design

**Fig. 1:**
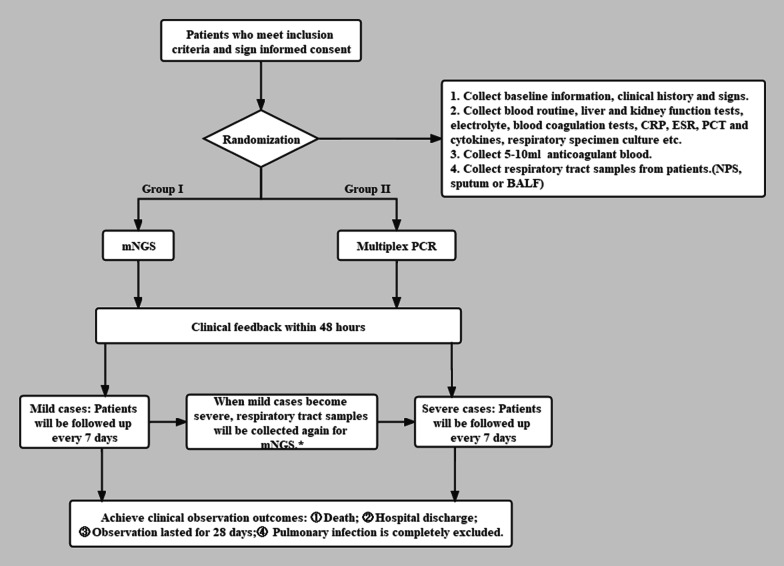
Study outline. *Group I will be detected by mNGS for pathogens. Group II will first receive respiratory multiplex PCR, then mNGS in severe pneumonia if multiplex PCR results are negative or inconsistent with the clinical conditions. *CRP* C-reactive protein test, *PCT* procalcitonin, *ESR* erythrocyte sedimentation rate, *NPS* nasopharynx swab, *BALF* bronchoalveolar lavage fluid, *mNGS* metagenomic next-generation sequencing

The study is a multicenter, open-label, randomized controlled, non-inferiority clinical trial recruiting patients with respiratory tract infections who meet inclusion and exclusion criteria (Table 1). Enrolled patients were divided into two groups in a 1:1 ratio [15]: Group I will be detected by mNGS for pathogens. Group II will first receive respiratory multiplex PCR, then mNGS in severe pneumonia (Table 2) if multiplex PCR results are negative or inconsistent with the clinical conditions. Disease condition and treatment status will be followed up every 7 days until reaching predefined outcomes within 28 days. If a participant with mild symptoms become severe during follow-up, respiratory samples should be collected again for mNGS detection. The detailed research process is shown in Fig. 1.

**Table 1:** Inclusion and exclusion criteria

Inclusion criteria	• Aged from 18 to 80 years old • Unlimited gender • Newly developed cough, expectoration, purulent sputum, or exacerbation of original respiratory diseases within 14 days • Meet at least one of the following items: (1) Fever (> 38 ℃) (2) Consolidation signs and/or wet rales on chest physical examination (3) Leucocyte > 10*10^9^/l or < 4*10^9^/l (4) Patchy infiltration or interstitial changes on chest radiology
Exclusion criteria	• Highly-suspected of or confirmed with noninfectious lung diseases (tumor, autoimmune diseases, etc.) • The pathogen has been identified • Insufficient respiratory tract or blood specimen • Unable or refusing to cooperate due to physical or psychological factors • Participating in other clinical studies • Investigators or clinicians considers unsafe for the subject to participate
Exit criteria	• Participants request to withdraw from the interview

**Table 2:** ATS/IDSA criteria for severe CAP

***Minor criteria***
Respiratory rate ≥ 30 breaths/min
PaO_2_/FiO_2_ ratio ≤ 250
Multilobar infiltration
Confusion/disorientation
Uremia (BUN ≥ 20 mg/dL)
Leukopenia (white blood cell count < 4 *10^9^/L)
Thrombocytopenia (platelet count < 100*10^9^/L)
Hypothermia (core temperature < 36 °C)
Hypotension requiring aggressive fluid resuscitation
***Major criteria***
Invasive mechanical ventilation
Septic shock with the need for vasopressors

Respiratory rate, a need for noninvasive ventilation can substitute for a respiratory rate > 30

PaO_2_/FiO_2_, arterial oxygen pressure/faction of inspired oxygen; BUN, blood urea nitrogen; Leukopenia, as a result of infection alone

### Ethical and confidential considerations

The study protocol and informed consent forms have been approved by the Ethical committee of Huashan Hospital affiliated to Fudan University (protocol ID: KY2021-450). The investigator will objectively and comprehensively introduce the purpose, procedure, potential benefits and risks of the study to the subjects and their legal representatives, and obtain informed consent through interview before registration. All patients will provide written informed consent.

### Site selection

**Fig. 2:**
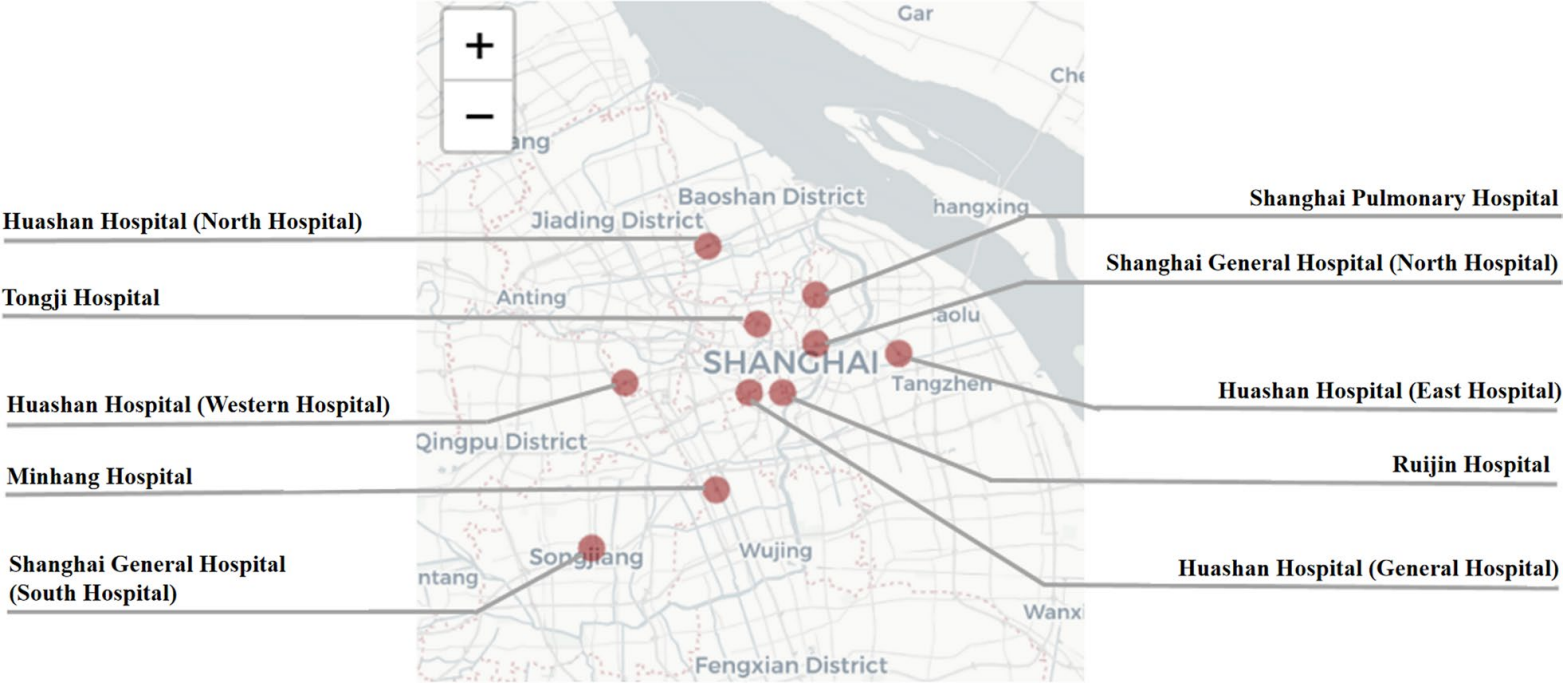
ESAR network. The map was acquired from: https://leafletjs.com/

This trial is led by the Huashan Hospital affiliated to Fudan University and the recruiting-cooperative units were distributed over 5 points in Shanghai: Huashan Hospital, Ruijin Hospital, Tongji Hospital, Shanghai Pulmonary Hospital, Shanghai General Hospital, Minhang Hospital and their branches. Project leader will conduct the quality control every six months. ESAR network for early identification of acute respiratory infection in Shanghai is shown in Fig. 2 [16].

### Study flowchart and clinical evaluation of enrolled participants

The following information will be collected at enrollment: baseline information, clinical history, physical examination, routine laboratory examinations (including blood routine, liver and kidney function test, electrolytes, blood coagulation tests, C reaction protein, erythrocyte sedimentation rate, procalcitonin, cytokines, etc.) and respiratory specimen culture. According to the above test results, the researchers evaluated the severity of pneumonia at the time of enrollment. 5–10ml EDTA anticoagulation and respiratory samples will be collected at enrollment (BALF ≥ 3 ml, or sputum ≥ 2 ml or more, or 2 nasopharyngeal swabs). After quality control, subjects will be randomly assigned to two groups according to the ratio of 1:1. Group I will be directly tested by mNGS, and group II will first receive multiplex PCR and subsequently mNGS according to multiplex PCR results and the severity of pneumonia. If mild pneumonia progressed to severity in subsequent follow-up, additional mNGS will be performed. Randomization was performed through an online central randomization system stratified by study site. Patients in both groups will be followed up for 28 days and a final diagnosis will be made by experienced physicians.

### Diagnostic methods

In this study, we plan to detect pathogens in group I by mNGS, and in group II by respiratory multiplex PCR. Reports will be returned within 48 hours. The later will include 16 respiratory pathogens and 3 atypical respiratory pathogens, including coronavirus, influenza A virus, influenza B virus, mycoplasma pneumoniae, adenovirus, respiratory syncytial virus parainfluenza virus (I, II, III, IV), human metapneumovirus, haemophilus influenzae, klebsiella pneumoniae, staphylococcus aureus. mNGS assays can simultaneously cover a wide spectrum of pathogens in a single sequence, including known pathogens and emerging unknown microorganisms.

### Duration of follow‑up

All participants will be followed up every 7 days for disease status and treatment conditions since enrollment, including symptoms, signs, routine laboratory examinations, along with respiratory specimen culture. Besides, SOFA score will be evaluated every 7 days.

### Sample size calculation

PASS 11 software was applied to calculate the sample size. Assuming hazard ratio (HR) = 1.50, *α* = 0.05, *β* = 0.10, and a 10% rate of participants in Group II receiving mNGS additionally, each group requires 200 subjects. Considering a 10% rate of lost to follow-up, each group needs 200 × 1.1 = 220 subjects; therefore, the total sample size of the two groups combined is calculated to be 440 subjects.

### Data collection and quality management

A series of quality assurance methods has been established and will be applied before and during ESAR project. All relevant personnel, including clinicians, laboratory personnel, and project management personnel will receive unified training on theoretical perspective and practical procedure.

On enrollment, clinicians are required to be familiar with the inclusion and exclusion criteria. Every case needs to be included continuously without selection or omission, and will be reviewed by the project leader from Huashan Hospital before official enrollment. On data collection, clinicians will be trained with filling case report form (CRF) before official enrollment. Questions in CRF should be filled face-to-face by clinicians during enrollment and should not be hinted, induced or recalled afterwards. All relevant forms should be properly kept in a suitable place by a designated person to prevent burning, tearing, smearing, etc.

To ensure that each center conducts research in strict accordance with the plan, and the data is authentic and credible, quality control is carried out by the research team of Huashan Hospital Affiliated to Fudan University every six months. Supervisors will be assigned by the Huashan Hospital affiliated to Fudan University.

### Adverse event management

Potential adverse events and their management will be evaluated during follow up. In this study, 8ml peripheral blood and respiratory tract samples will be collected along with the routine clinical examination, which will not cause additional operations, burdens and costs to the subjects. Potential adverse events include localized pain, bruising or needle infections.

If a patient has any psychological discomfort in the process, the clinician should provide timely comfort and notify the relevant personnel to evaluate and record within 2 h. If ineffective, a psychologist will be arranged to conduct psychological assessment and consultation within 12 h, which will be recorded by designated personnel. The subjects can receive corresponding compensation and treatment when an unexpected injury or loss related to the research come out. Any adverse events in this study will be recorded in detail by a dedicated person. Clinicians must carefully inquire and trace any adverse events during the trial, strictly record them in the CRF, and finally report them to the ethics committee and relevant departments.

### Assessment and analysis of outcomes

The primary and secondary outcomes will be compared between group I and group II. Primary outcomes are time to start targeted antibiotic therapy. Secondary outcomes include incidence of clinically significant events (oxygen inhalation, ICU admission, tracheal intubation, tracheostomy, death, etc.), clinical remission rate (Meet all the following criteria for at least 24 h: (1) Heart rate < 100 bpm; (2) Blood pressure > 90 mmHg; (3) Body temperature < 38 °C; (4) Respiratory rate < 24/min; (5) Oxygen saturation at room temperature > 90%), hospitalization length within 28-day follow up.

SPSS 20 and Prism 8.0 will be applied for statistical analysis. Firstly, whether the enrolled cases met inclusion and exclusion criteria, and whether the actual number of enrolled, excluded, and dropped cases in each center are counted will be reviewed at the end of the study. Second, demographics characteristics, baseline information, follow-up status and outcomes will be compared between two groups. Statistical methods including *t* test or Mann–Whitney test, Pearson's chi-square test or Fisher test, cox method, Kaplan-Meier survival curve, log-rank method, logistic regression models will be applied in the form of two-tailed test. *P* < 0.05 was considered statistically significant.

### Confidentiality

Participants’ personal information is strictly restricted to outcome evaluation of study regimen. Paper documents containing participants information will be saved in a dedicated office in cooperative hospitals. Digital documents will be kept in password-protected files on website. The study documents can only be accessed by authorized personnel.

## Discussion

Exploring the efficiency of different diagnosis strategies is of great significance for optimizing ARI early management and prognosis. Since multiplex PCR was first applied by Chamberlian et al. to screen missing locus on Duchenne muscular dystrophy in 1988 [17], it has been successfully applied in many fields. The sensitivity and specificity of respiratory multiplex PCR has been proved to be higher than 90% [18]. Shengchen et al. [19] reported that FA-RP can shorten the length of stay and reduce antibiotic administration in hospitalized adults with lower respiratory tract infection. Similarly, our previous study [20] found that 53.8% received antiviral therapy and 69.6% received antibiotics adjustment in multiplex PCR group, while 12.7% and 5.1% in traditional examination group, indicating a positive effect on outcomes. Simultaneously, FA-RP can also lower the risk of infectious disease transmission [20]. However, Brendish et al. [21] argued that multiplex PCR was not associated with a reduction in the overall duration of antibiotic use in hospitalized adults with acute respiratory illness. Besides, multiplex PCR cannot detect rare or unknown respiratory pathogens.

Compared with traditional culture, mNGS improves sensitivity and shortens time to identify various pathogens [22]. The sensitivity and specificity in BALF samples were 88.89% and 14.86% [23], and 34% of candidate pathogens can be detected by mNGS [24] to provide more accurate diagnostic information. Furthermore, it can screen rare pathogens [25] such as COVID-19 [26] and Nocardia [27], but subject to missing detection, expensive equipment and high personnel demand. There is few studied reporting impact of mNGS on ARI prognosis. Zhang et al. [28] performed repeated mNGS testing in nine patients with suspected central nervous system infection (CNS) and found that mNGS semi-quantitative values can be used to dynamically monitor pathogen load and disease progression. Hongxia Duan et al. [29] suggested that 28-day mortality in mNGS-positive group was higher (9.0% vs 0%, *P *= 0.049), but the average survival time (176.64 vs 150.96 days, *P *= 0.425) was not statistically different, indicating that positive results were correlated with worse prognosis. However, these studies did not analyze the influence of antibiotics, which might lead to the lower detection rate by traditional methods.

Although mNGS can cover most pathogens, it requires longer time than respiratory multiplex PCR. Moreover, the expensive cost makes mNGS not a panacea for any respiratory infection, but a technology that can be used in specific situations. Therefore, based on previous research, ESAR is designed to compare a combination of respiratory multiplex PCR and mNGS with direct mNGS detection to optimize diagnosis pathway in ARI.

This study has several limitations. Firstly, although multiplex PCR and mNGS will be performed in the central laboratory, there existed certain heterogeneity for traditional respiratory specimen detection method between hospitals. In addition, all the methods above still cannot directly distinguish pathogenic organism and colonization. The reports will be interpreted by experienced clinicians and controversial cases will be discussed and diagnosed by experts.

## Data Availability

Not applicable.

## Abbreviations

WHO: World Health Organization
LRTI: Lower respiratory tract infection
qPCR: Real-time quantitative PCR
RT-PCR: Reverse transcription PCR
dPCR: Digital PCR
ddPCR: Digital droplet PCR
FA-RP: Filmarray respiratory detection system
NPS: Nasopharyngeal swab
TB: Tuberculosis
MTB: Mycobacterium tuberculosis
RIF: Rifampicin
POCT: Real-time checkout
ARIs: Acute respiratory infections
mNGS: Next generation metagenomic sequencing
PCR: Polymerase chain reaction
CRF: Case report form
COVID-19: Novel coronavirus pneumonia
H1N1: Swine-origin influenza A virus
BALF: Bronchoalveolar fluid
HR: Hazard ratio
CNS: Nervous system infection

## References

[1] Global burden of 369 diseases and injuries in 204 countries and territories, 1990–2019: a systematic analysis for the Global Burden of Disease Study 2019. Lancet*.* 2020;396(10258):1204–1222. 10.1016/S0140-6736(20)30925-9 PMC7567026 33069326

[2] Wiersinga WJ, Rhodes A, Cheng AC, Peacock SJ, Prescott HC (2020). Pathophysiology, transmission, diagnosis, and treatment of coronavirus disease 2019 (COVID-19): a review. JAMA.

[3] Feng L, Feng S, Chen T (2020). Burden of influenza-associated outpatient influenza-like illness consultations in China, 2006–2015: a population-based study. Influenza Other Respir Viruses.

[4] Global Health Estimates: life expectancy and leading causes of death and disability. http://www.who.int/healthinfo/global_burden_disease/estimates/en/index1.html. Accessed 11 May 2022.

[5] Woodhead M, Welch CA, Harrison DA, Bellingan G, Ayres JG (2006). Community-acquired pneumonia on the intensive care unit: secondary analysis of 17,869 cases in the ICNARC Case Mix Programme Database. Crit Care.

[6] Poole S, Clark TW (2020). Rapid syndromic molecular testing in pneumonia: the current landscape and future potential. J Infect.

[7] Poritz MA, Blaschke AJ, Byington CL (2011). FilmArray, an automated nested multiplex PCR system for multi-pathogen detection: development and application to respiratory tract infection. PLoS ONE.

[8] Mahony JB (2010). Nucleic acid amplification-based diagnosis of respiratory virus infections. Expert Rev Anti Infect Therapy.

[9] Kaku N, Hashiguchi K, Iwanaga Y (2018). Evaluation of FilmArray respiratory panel multiplex polymerase chain reaction assay for detection of pathogens in adult outpatients with acute respiratory tract infection. J Infect Chemother.

[10] Leber AL, Lisby JG, Hansen G (2020). Multicenter evaluation of the QIAstat-Dx respiratory panel for detection of viruses and bacteria in nasopharyngeal swab specimens. J Clin Microbiol.

[11] Huang HS, Tsai CL, Chang J, Hsu TC, Lin S, Lee CC (2018). Multiplex PCR system for the rapid diagnosis of respiratory virus infection: systematic review and meta-analysis. Clin Microbiol Infect.

[12] Qian Y-Y, Wang H-Y, Zhou Y (2020). Improving pulmonary infection diagnosis with metagenomic next generation sequencing. Front Cell Infect Microbiol.

[13] Ramachandran PS, Wilson MR (2020). Metagenomics for neurological infections—expanding our imagination. Nat Rev Neurol.

[14] Zhu N, Zhang D, Wang W (2020). A novel coronavirus from patients with pneumonia in China, 2019. N Engl J Med.

[15] Mandell LA, Wunderink RG, Anzueto A (2007). Infectious Diseases Society of America/American Thoracic Society consensus guidelines on the management of community-acquired pneumonia in adults. Clin Infect Dis.

[16] Leaflet. https://leafletjs.com/.

[17] Chamberlain JS, Gibbs RA, Ranier JE, Nguyen PN, Caskey CT (1988). Deletion screening of the Duchenne muscular dystrophy locus via multiplex DNA amplification. Nucleic Acids Res.

[18] Murphy CN, Fowler R, Balada-Llasat JM (2020). Multicenter evaluation of the BioFire FilmArray pneumonia/pneumonia plus panel for detection and quantification of agents of lower respiratory tract infection. J Clin Microbiol.

[19] Shengchen D, Gu X, Fan G (2019). Evaluation of a molecular point-of-care testing for viral and atypical pathogens on intravenous antibiotic duration in hospitalized adults with lower respiratory tract infection: a randomized clinical trial. Clin Microbiol Infect.

[20] Qian Y, Ai J, Wu J (2020). Rapid detection of respiratory organisms with FilmArray respiratory panel and its impact on clinical decisions in Shanghai, China, 2016–2018. Influenza Other Respir Viruses.

[21] Brendish NJ, Malachira AK, Armstrong L (2017). Routine molecular point-of-care testing for respiratory viruses in adults presenting to hospital with acute respiratory illness (ResPOC): a pragmatic, open-label, randomised controlled trial. Lancet Respir Med.

[22] Schlaberg R, Queen K, Simmon K (2017). Viral pathogen detection by metagenomics and pan-viral group polymerase chain reaction in children with pneumonia lacking identifiable etiology. J Infect Dis.

[23] Chen Y, Feng W, Ye K (2021). Application of metagenomic next-generation sequencing in the diagnosis of pulmonary infectious pathogens from bronchoalveolar lavage samples. Front Cell Infect Microbiol.

[24] Miao Q, Ma Y, Wang Q (2018). Microbiological diagnostic performance of metagenomic next-generation sequencing when applied to clinical practice. Clin Infect Dis.

[25] Chen H, Yin Y, Gao H (2020). Clinical utility of in-house metagenomic next-generation sequencing for the diagnosis of lower respiratory tract infections and analysis of the host immune response. Clin Infect Dis.

[26] Mostafa HH, Fissel JA, Fanelli B, et al. Metagenomic next-generation sequencing of nasopharyngeal specimens collected from confirmed and suspect COVID-19 patients. mBio*.* 2020;11(6). 10.1128/mBio.01969-20 PMC7686804 33219095

[27] Weng S-S, Zhang H-Y, Ai J-W (2020). Rapid detection of by next-generation sequencing. Front Cell Infect Microbiol.

[28] Zhang Y, Cui P, Zhang H-C (2020). Clinical application and evaluation of metagenomic next-generation sequencing in suspected adult central nervous system infection. J Transl Med.

[29] Duan H, Li X, Mei A (2021). The diagnostic value of metagenomic next⁃generation sequencing in infectious diseases. BMC Infect Dis.

